# Unrecognized Fibrosis Risk in MASLD: A Real-World Analysis and the Case for AI-Augmented Stratification

**DOI:** 10.1016/j.gastha.2025.100857

**Published:** 2025-12-01

**Authors:** Ruona Ebiai, Jasmine McNair, Sameera Shuaibi, Adil Memon, Anshuman Desai, Lisa Birdsall Fort, Leo Seoane, Nigel Girgrah, George Therapondos

**Affiliations:** 1 Department of Internal Medicine, Ochsner Clinic Foundation, New Orleans, Louisiana; 2 Department of Gastroenterology and Hepatology, Ochsner Clinic Foundation, New Orleans, Louisiana; 3 Ochsner Center for Innovation, New Orleans, Louisiana

**Keywords:** MASLD, Hepatic steatosis, Fibrosis risk, FIB-4, Artificial intelligence, Liver disease

## Abstract

**Background and Aims:**

Current fibrosis risk stratification in metabolic dysfunction–associated steatotic liver disease (MASLD) relies on provider-initiated noninvasive testing and referral, making it vulnerable to variability in awareness, documentation, and follow-through. We aimed to quantify care gaps associated with this provider-dependent approach and explore opportunities for artificial intelligence to improve MASLD detection and management.

**Methods:**

We performed a retrospective analysis of all adults undergoing abdominal ultrasound in 2024 at Ochsner Health’s South Shore campuses. Natural language processing identified reports with hepatic steatosis, and patients with at least 1 cardiometabolic risk factor were included. Fibrosis-4 index (FIB-4) scores were calculated from recent laboratory data (within 6 months of ultrasound) using age-adjusted thresholds to classify patients as low, indeterminate, or high fibrosis risk. Management was defined as hepatology referral for high- or indeterminate-risk patients and documentation of a primary care provider for low-risk patients requiring reassessment.

**Results:**

Among 14,814 adults with ultrasound in 2024, 3052 (20.6%) met the MASLD criteria. Based on age-adjusted FIB-4, 15.2% were high risk, 18.0% indeterminate, and 66.0% low risk for advanced fibrosis. Of 465 high-risk patients, only 33.5% had hepatology referrals, leaving 309 (10.1% of the MASLD cohort) without appropriate specialty evaluation. Among 549 indeterminate-risk patients, 58.7% lacked referral for secondary assessment. In the low-risk group, 224 (7.3%) had no documented primary care provider for follow-up, and 24 (0.8%) lacked sufficient laboratory data for FIB-4 calculation. Overall, 28.0% of the MASLD cohort had a critical, moderate, or monitoring care gap.

**Conclusion:**

Significant gaps persist in MASLD fibrosis risk stratification and management, largely reflecting system-level coordination failures rather than access barriers. Artificial intelligence–driven workflows integrated into the electronic health record could automate steatosis detection, calculate FIB-4 scores, flag care gaps, and prompt risk-stratified referrals or reassessments, offering a scalable solution to standardize MASLD management and improve outcomes.

## Introduction

Metabolic dysfunction–associated steatotic liver disease (MASLD) is the fastest-growing global contributor to liver disease, with an estimated prevalence of ∼30%.^1^ In the United States, it is rapidly becoming the leading indication for liver transplantation.^2^ This trend is largely driven by the global rise in obesity and type 2 diabetes mellitus, both major risk factors for MASLD.^3^ For many patients, MASLD remains a silent disease, often undiagnosed until advanced fibrosis or cirrhosis has developed, making early identification and risk stratification critical to prevent disease progression.^4^ Given the impracticality of liver biopsy for widespread screening, noninvasive tests (NITs), such as the fibrosis-4 index (FIB-4), have been validated as tools to identify patients at risk for advanced fibrosis who may benefit from hepatology referral. However, the uptake of NITs in routine clinical practice remains inconsistent.^5^^,^^6^ Multiple barriers may contribute to suboptimal care for patients with hepatic steatosis, including limited awareness of MASLD progression, variable adoption of NITs, and delays or errors introduced by manual review of imaging reports and labs.^7^^,^^8^ As a result, many patients may be inadequately risk-stratified: low-risk individuals may not be re-evaluated, those at indeterminate risk may miss timely secondary assessment, and high-risk patients may fail to receive appropriate specialty management or follow-up. In this study, we aimed to quantify care gaps in MASLD fibrosis risk stratification at our institution and evaluate the potential role of artificial intelligence (AI)–augmented clinical workflows to address them. We hypothesized that the current provider-dependent, manual risk stratification process leads to systematic underidentification of at-risk patients, increasing the likelihood of missed diagnoses, delayed referrals, and progression of disease.

## Methods

### Study Design and Setting

We conducted a retrospective descriptive analysis using electronic health record (EHR) data from all adult patients who underwent abdominal ultrasound imaging in 2024 at Ochsner Health System's South Shore campuses in the Greater New Orleans area.

### Initial Study Population

A total of 14,814 unique adult (≥18 years of age) patients who underwent abdominal ultrasound imaging between January 1, 2024, and December 31, 2024, were identified from the EHR database.

### Inclusion/Exclusion Criteria

#### Inclusion criteria

This study included adult patients aged 18 years and older who underwent abdominal ultrasound in 2024. Eligible patients were required to have ultrasound reports containing terminology consistent with hepatic steatosis, as identified through natural language processing (NLP). Additionally, patients were required to have at least 1 documented cardiometabolic risk factor consistent with MASLD, which for this study included overweight or obesity (body mass index (BMI) ≥25 kg/m^2^), hypertension, diabetes mellitus, or hyperlipidemia.

#### Exclusion criteria

Patients were excluded if they were younger than 18 years, had ultrasound reports without evidence of hepatic steatosis, or had evidence of hepatic steatosis but no documented cardiometabolic risk factors consistent with MASLD.

### Final Study Population

From the initial cohort of 14,814 patients–3256 patients (22.0%) had evidence of hepatic steatosis on ultrasound. Hepatic steatosis was identified using NLP techniques applied to free-text ultrasound reports. A comprehensive keyword-based search algorithm was employed to detect the presence of hepatic steatosis terminology, including: "fatty liver," "hepatic steatosis," "steatosis," "fatty infiltration," "increased hepatic echogenicity," "increased hepatic echotexture," "echogenic liver," "bright liver," "fatty change," and "hepatic fat." The search was performed using case-insensitive string matching across all ultrasound report narratives.

Of these patients with identified evidence of hepatic steatosis, 3052 patients (93.7%) met the inclusion criteria by having at least 1 cardiometabolic risk factor consistent with MASLD, representing the final study cohort for analysis.

### Patient Categorization and Grouping

The 3052 patients (20.6%) whose ultrasound reports indicated the presence of hepatic steatosis with at least one cardiometabolic risk factor consistent with MASLD were identified for additional analysis. These patients were subsequently stratified by risk of disease progression based on their most recent available FIB-4 score and assessed for receipt of guideline-based care^9^ corresponding to their risk category, including secondary risk assessment, referral to a hepatology specialist, or documentation of a primary care provider (PCP) for ongoing re-evaluation (Figure and Table 1).

**Figure:**
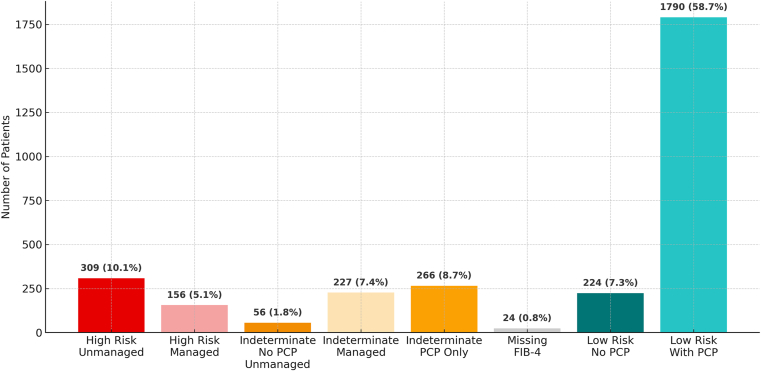
Patient grouping by fibrosis risk and management status.

**Table 1:** Patient Grouping by Fibrosis Risk and Management Status (N = 3052)

Group	Description	Count (n, %)
A (high risk, unmanaged)	FIB-4 >2.67; no hepatology referral documented	309 (10.1%)
B (high risk, managed)	FIB-4 >2.67; hepatology referral documented	156 (5.1%)
C (indeterminate, No PCP, unmanaged)	Indeterminate FIB-4; no PCP; no referral documented	56 (1.8%)
D (indeterminate, managed)	Indeterminate FIB-4; hepatology referral documented	227 (7.4%)
E (indeterminate, unmanaged)	Indeterminate FIB-4; PCP documented but no referral documented	266 (8.7%)
F (missing FIB-4)	Insufficient or outdated labs to calculate FIB-4	24 (0.8%)
G (low risk, No PCP)	FIB-4 below threshold; no documented PCP	224 (7.3%)
H (low risk, with PCP)	FIB-4 below threshold; PCP documented	1790 (58.7%)

### Definitions of Categories

Patients were categorized using the following definitions.

#### FIB-4 risk stratification thresholds

Fibrosis risk stratification was determined using age-adjusted FIB-4 thresholds. For individuals younger than 65 years, low risk was defined as FIB-4 <1.3, indeterminate risk as 1.3–2.67, and high risk as >2.67. For those aged 65 years and older, low risk was defined as FIB-4 <2.0, indeterminate risk as 2.0–2.67, and high risk as >2.67.

#### Management status

Management status was defined based on hepatology referral documentation in the electronic medical record (EMR). Patients were considered “managed” if any hepatology referral had been recorded at any time and “unmanaged” if no such referral existed. A PCP was considered present if a named PCP was documented in the EMR. Cases with insufficient recent laboratory data, specifically missing aspartate aminotransferase (AST), alanine aminotransferase (ALT), or platelet count, were classified as “missing FIB-4.”

Based on these criteria, patients were assigned to 1 of 8 categories: group A included high-risk patients without hepatology referral; group B included high-risk patients with hepatology referral; group C represented indeterminate-risk patients without a documented PCP or hepatology referral; group D comprised indeterminate-risk patients with hepatology referral; group E represented indeterminate-risk patients with a documented PCP but no hepatology referral; group F included patients with missing laboratory data precluding FIB-4 calculation; group G included low-risk patients without a documented PCP; and group H comprised low-risk patients with a documented PCP.

### Data Analysis

#### Data extraction and processing

Data were extracted from EPIC© (EPIC Systems Corporation, Verona, WI) EHR system. NLP algorithms (via Regular expressions aka REGex) were used to identify hepatic steatosis terminology within ultrasound reports and extract relevant clinical variables from structured and unstructured data fields. Clinical, laboratory, demographic, and referral data were obtained across ambulatory and inpatient settings.

#### Data validation

To ensure data accuracy, a subset of patient records was randomly selected for manual chart review. Key variables, including clinical characteristics, laboratory values, referral documentation, and listed PCP, were cross-checked against the EHR data. No discrepancies were identified.

#### Laboratory data and FIB-4 calculation

FIB-4 scores were calculated using the most recent available laboratory values for AST, ALT, platelets, and patient age (at the time of available lab) according to the standard formula: FIB-4 = (Age × AST)/(Platelets × √ALT). Of the 3052 patients in the MASLD cohort, 3028 (99.2%) had complete laboratory data (platelets, ALT, AST, and age at time of labs) available for FIB-4 calculation. Most laboratory values were recent, with approximately 96% obtained in 2024 or later. To ensure clinical relevance, FIB-4 scores were calculated using laboratory values collected within 6 months before or after the qualifying abdominal ultrasound. All labs were obtained prior to data analysis in June 2025.

### Statistical Analysis

Descriptive statistics were calculated for all variables. Continuous variables are reported as means with standard deviations or medians with interquartile ranges, depending on distribution. Categorical variables are reported as counts and percentages. Group comparisons were performed using chi-square or Fisher’s exact tests for categorical variables, and t-tests or Mann–Whitney U tests for continuous variables where appropriate. A 2-sided *P* value <.05 was considered statistically significant. All analyses were conducted using Python 3.11.6 with Pandas 2.2.3 and SciPy 1.11.3 libraries. Analyses were performed on data extracted in June 2025, 6 months after the last eligible ultrasound. This interval was chosen to allow adequate time for referrals to be placed and documented in the EHR.

### Ethical Considerations

This study was reviewed by the Institutional Review Board of Ochsner Health system which determined it did not constitute human subjects' research.

## Results

### Demographics

The study cohort included 3052 adults with MASLD (Table 2). The mean age was 55.2 years (standard deviation ± 15.9), and the median age was 57.0 years. Age distribution was as follows: 19.2% (n = 587) were 18–39 years, 37.6% (n = 1147) were 40–59 years, and 43.1% (n = 1315) were ≥60 years.

**Table 2:** Demographic and Clinical Characteristics of the Study Cohort (N = 3052)

Characteristic	n (%)
Age	
18–39 y	587 (19.2)
40–59 y	1147 (37.6)
≥60 y	1315 (43.1)
Mean ± standard deviation, y	55.2 ± 15.9
Median (interquartile range), y	57 (43–67)
Sex	
Male	1559 (51.1)
Female	1493 (48.9)
Race	
White	1990 (65.2)
Black or African American	769 (25.2)
Asian	106 (3.5)
Other	109 (3.6)
Multi race	43 (1.4)
American Indian or Alaska Native	13 (0.4)
Unknown/Refused	22 (0.7)
Ethnicity	
Not Hispanic or Latino/a	2735 (89.6)
Hispanic or Latino/a	304 (10.0)
Unknown/Refused	13 (0.4)
BMI	
Normal weight (18.5–24.9 kg/m^2^)	333 (10.9)
Overweight (25.0–29.9 kg/m^2^)	874 (28.6)
Obesity class I (30.0–34.9 kg/m^2^)	872 (28.6)
Obesity class II (35.0–39.9 kg/m^2^)	503 (16.5)
Obesity class III (≥40.0 kg/m^2^)	448 (14.7)
Overweight/Obesity (≥25 kg/m^2^)	2697 (88.4)
Comorbidities	
Hypertension	1983 (65.0)
Diabetes mellitus	1096 (35.9)
Hyperlipidemia	1866 (61.1)
Multiple risk factors (≥2)	1673 (54.8)
FIB-4 risk category	
Low risk (<1.3)	2014 (66.0)
Indeterminate risk (1.3–2.67)	549 (18.0)
High risk (>2.67)	465 (15.2)

The cohort was 51.1% male (n = 1559) and 48.9% female (n = 1493). Most patients were White (65.2%, n = 1990), followed by Black or African American (25.2%, n = 769), with smaller proportions identifying as Asian (3.5%) or other races. Approximately 10.0% (n = 304) were Hispanic or Latino/a.

The mean BMI was 32.7 kg/m^2^ (median 31.6 kg/m^2^), with 88.4% (n = 2697) classified as overweight or obese (BMI ≥25 kg/m^2^). Hypertension was present in 65.0% (n = 1983) of patients, diabetes mellitus in 35.9% (n = 1096), and hyperlipidemia in 61.1% (n = 1866). Notably, 54.8% (n = 1673) had 2 or more cardiometabolic risk factors.

Based on age-adjusted FIB-4 scores, 66.0% (n = 2014) of patients were classified as low risk, 18.0% (n = 549) as indeterminate risk, and 15.2% (n = 465) as high risk for advanced fibrosis.

### Categorization of Patients by Fibrosis Risk and Management Status

Of the 3052 patients categorized, 10.1% (n = 309) were high risk without a documented hepatology referral (group A), and 5.1% (n = 156) were high risk with a referral (group B).

Among patients at indeterminate risk, 1.8% (n = 56) had no PCP or referral documented (group C), 7.4% (n = 227) were managed with a hepatology referral (group D), and 8.7% (n = 266) had a documented PCP but no referral (group E).

Patients lacking sufficient laboratory data to calculate FIB-4 comprised 0.8% (n = 24) (Group F).

Among low-risk patients, 7.3% (n = 224) had no documented PCP (group G), while 58.7% (n = 1790) had an identified PCP (group H).

This distribution (Figure and Table 1) highlights key care gaps, notably the substantial proportion of high-risk patients lacking specialty referral and indeterminate-risk patients without clear pathways for secondary risk assessment or re-evaluation.

At our institution, secondary fibrosis risk assessment e.g. vibration-controlled transient elastography (VCTE) is primarily performed by hepatology and not PCP, thus any patient without specialist referral is unlikely to have had a secondary assessment of their indeterminate FIB-4/possible fibrosis.

### Critical Management Gaps in High-Risk Patients

Of 465 patients classified as high-risk and requiring hepatology evaluation, only 156 (33.5%) had a documented hepatology referral, leaving 309 patients (66.5% of high-risk cases; 10.1% of the total cohort) without appropriate specialist management (group A). This represents the most critical care gap, as these patients have the greatest probability of advanced fibrosis and disease progression.

Importantly, this gap cannot be attributed to lack of health care engagement as 77.0% of unmanaged high-risk patients had established PCPs, compared with 91.7% of managed patients (*P* < .001), possibly indicating missed opportunities for referral initiation at the primary care level.

### Suboptimal Management of Indeterminate-Risk Patients

Indeterminate-risk patients comprised 18.0% of the cohort (n = 549), yet 58.7% (n = 322) lacked hepatology referral for guideline-recommended secondary risk assessment (eg, VCTE). The largest gap was in group E, which included 266 patients (48.5% of indeterminate cases) who had established primary care but no specialty referral.

### Health Care Engagement Paradox

The analysis revealed a paradoxical relationship between health care engagement and specialist management. Among all patients requiring hepatology evaluation (high-risk and indeterminate combined), 79.2% had documented PCPs, yet only 38.3% received appropriate specialty referrals. These findings challenge assumptions that management gaps result primarily from patient access barriers and instead point to systemic issues in care coordination and referral patterns within established health care relationships.

### Reassessment Opportunities in Low-Risk Patients and Resource Allocation

Low-risk patients make up the largest portion of the MASLD cohort (66.0%, n = 2014), offering a significant opportunity for reassessment protocols to prevent disease progression.

Although currently classified as low risk, 38.0% (n = 766) have FIB-4 scores >1.0, nearing the indeterminate threshold, thus further emphasizing the need for structured longitudinal monitoring. With 88.9% having established primary care relationships, this group is well-suited for EMR-based care gap alerts and automated reassessment workflows.

However, resource allocation patterns merit review: 32.0% of low-risk patients (n = 645) already have hepatology referrals. This includes 233 patients with “very low” FIB-4 scores (≤0.8).

While some of these referrals may have been initiated for non-MASLD reasons, this pattern suggests opportunities to optimize specialty care utilization and focus hepatology resources on patients with clear fibrosis risk in MASLD.

In general, the size of the low-risk population (n = 2014) highlights the need for EMR-driven surveillance systems that automatically flag patients for periodic reassessment, particularly those with diabetes or significant metabolic risk factors, while preserving specialty care capacity for higher-risk individuals.

### Management Challenges in Young Adults

Patients younger than 35 years comprised a small but clinically important subset of the MASLD cohort, accounting for 11.5% (n = 350). Risk stratification using FIB-4 in this population is inherently limited, as the score lacks validation in individuals ≤35 years, making standard algorithms less reliable for clinical decision-making. Despite this limitation, FIB-4 calculations classified 90.6% as low-risk, 4.6% as indeterminate-risk, and 4.6% as high-risk, though these classifications should be interpreted cautiously given the score’s poor performance characteristics in younger patients.^10^

Hepatology referral rates among those needing referral (high or indeterminate risk) were lower in patients <35 years compared to those ≥35 years (18.8% vs 39.2%). Within the younger subgroup, 6 of 32 patients (18.8%) had documented hepatology referrals, leaving 26 (81.2%) without specialty evaluation. This under-referral is concerning, as young adults with suspected MASLD require alternative fibrosis assessment modalities such as VCTE or magnetic resonance elastography, which aren’t typically implemented in primary care settings.

Notably, 75.4% of young patients (less than age 35) had established primary care relationships, highlighting an opportunity to improve screening and referral strategies. However, reliance on FIB-4 alone is inappropriate in this age group.

These findings underscore the need for age-specific MASLD management protocols that account for the diagnostic limitations of FIB-4 in younger adults while ensuring timely referral for comprehensive evaluation and alternative risk assessment strategies.

### Transaminase Patterns and Clinical Implications

Transaminase elevations (ALT >40 or AST >40 U/L) were observed in 1083 patients (35.5%) of the MASLD cohort. Among high-risk patients (FIB-4 >2.67), 144 patients (31.0%) had normal transaminases (ALT ≤40 and AST ≤40 U/L), indicating that normal aminotransferase levels do not exclude advanced fibrosis.

### Overall Care Quality Metrics

Within the total MASLD cohort (n = 3052), 855 patients (28.0%) were classified as inadequately managed according to guideline-based standards. This group comprised 309 high-risk patients (10.1%) without documented hepatology referrals, 322 indeterminate-risk patients (10.6%) lacking appropriate secondary risk assessment, 224 low-risk patients (7.3%) without a PCP for routine reassessment, and 24 patients (0.8%) with insufficient laboratory data for FIB-4 calculation and risk stratification.

Management gaps were further stratified by clinical priority. Critical gaps referred to high-risk patients without a documented hepatology referral (n = 309; 10.1%). Moderate gaps included indeterminate-risk patients who had not undergone secondary fibrosis risk assessment (n = 322; 10.6%). Monitoring gaps described low-risk patients without an established PCP for longitudinal follow-up (n = 224; 7.3%). Finally, assessment gaps included patients with incomplete laboratory data, precluding calculation of a FIB-4 score (n = 24; 0.8%). These findings reveal a continuum of care deficiencies, with critical gaps affecting approximately 1 in 10 high-risk patients, a group requiring urgent specialist evaluation due to their elevated probability of advanced fibrosis.

The 631 patients (20.7%) with critical and moderate management gaps represent the highest-priority population for quality improvement initiatives, including EMR-based clinical decision support tools and streamlined referral pathways to reduce progression risk and optimize specialist resource utilization.

## Discussion

In this analysis of 3052 MASLD patients, 855 (28.0%) were identified as inadequately managed according to evidence-based guidelines. Notably, 631 patients (20.7%) had high-priority gaps, including high-risk patients without hepatology referrals and indeterminate-risk patients lacking advanced risk stratification. While 2197 patients (72.0%) received care consistent with current recommendations, the persistent gaps highlight opportunities for quality improvement initiatives.

Primary care engagement was high (87.5%), suggesting that suboptimal care coordination rather than access is the primary barrier to appropriate MASLD management. Strengthening referral pathways between primary care and hepatology services is essential to prevent progression of fibrosis among high-risk and indeterminate-risk patients.

Our transaminase analysis revealed that 31.0% of high-risk patients had normal aminotransferase levels, and 59.0% of these patients lacked hepatology referrals. This underscores the limitations of transaminase-based screening, which may miss advanced fibrosis in patients without overt biochemical abnormalities. These findings support the need for systematic FIB-4–based risk stratification rather than relying solely on enzyme elevations.

These findings are consistent with prior observations. Leung et al. reported that only a small fraction of high-risk nonalcoholic fatty liver disease patients, particularly those with diabetes, were referred to hepatology, while most referrals occurred in patients with low FIB-4 scores.^11^ Similar to our results, their study emphasized that referral failures were related to workflow limitations and provider awareness rather than patient access. This supports the need for system-level and AI-assisted approaches to improve linkage to care and ensure appropriate management of patients at risk for advanced fibrosis.

Although this analysis was conducted within a single health system, it used a real-world, all-comers approach that included all adults undergoing abdominal ultrasound across multiple campuses. The prevalence of MASLD in our cohort was consistent with national estimates, supporting representativeness. These findings are therefore unlikely to be unique to our institution. Similar care gaps have been documented across diverse health care systems,^11^ reflecting a broader limitation of provider-dependent workflows. While institutions with dedicated MASLD clinics or greater frontline awareness may achieve modestly higher referral rates, it is improbable that any center achieves complete identification and management of all at-risk patients. Thus, the observed deficiencies likely reflect a systemic challenge that warrants scalable, automated solutions to standardize MASLD risk stratification and linkage to care.

### Limitations

Our study has limitations. The initial data pull included over 14,000 patients, but not all charts were manually verified, which may have introduced misclassification or incomplete capture of care details. Care provided outside our health system may not have been recorded. The study relied on radiologist ultrasound reads and individual imaging studies were not manually verified.

Additionally, our analysis included diabetes mellitus but not prediabetes, potentially underestimating the prevalence of MASLD and related risk factors. Our definition of dyslipidemia was limited to documented hyperlipidemia, which may also underestimate MASLD prevalence by excluding other components of dyslipidemia such as low high-density lipoprotein or elevated triglycerides. Fibrosis risk is multifactorial, and other etiologies such as alcohol-associated liver disease were not systematically excluded.

### Operational Considerations and Institutional Capacity for Scaled MASLD Management

We recognize that systematic FIB-4 screening across our health system would identify a substantial number of patients with non-normal results, which could initially exceed the capacity of our hepatology service to evaluate all high- or indeterminate-risk individuals. At present, our institution does not routinely offer enhanced liver fibrosis testing, and VCTE is typically performed within hepatology clinics rather than as a point-of-care test. Institutional stakeholders are actively exploring scalable solutions to address this limitation. Given that the majority of MASLD patients in our cohort are already engaged with primary care, frontline management and reassessment of low- and indeterminate-risk groups within primary care is both feasible and essential. Collaborative work is underway with endocrinology and population health leadership to leverage the diabetic registry and integrate risk-based screening strategies that prioritize high-risk MASLD patients for hepatology referral. Over time, this multidisciplinary model aims to expand access, balance specialty capacity, and establish a sustainable pathway for comprehensive MASLD identification and management across the health system.

### AI-Assisted Clinical Decision Support

The complexity of MASLD risk stratification, combined with manual screening limitations, supports the implementation of AI-assisted clinical workflows. Automated systems could: identify hepatic steatosis from imaging reports, calculate FIB-4 scores using available laboratory data and generate real-time evidence-based recommendations for risk-stratified management. Such systems would reduce missed cases of advanced fibrosis due to normal transaminases, standardize risk assessment, and facilitate timely referrals.

### Workflow Integration

With high primary care engagement, AI-generated care gap alerts could be effectively deployed within EHRs. These alerts could: prompt hepatology referral for appropriate high-risk and indeterminate-risk patients and establish automated monitoring schedules for low-risk individuals. This proactive approach could shift MASLD care from reactive, symptom-driven workflows to structured, risk-based management, ensuring consistent application of guidelines across diverse care settings.

### Future Directions

Prospective studies evaluating AI-driven decision support tools are needed to determine clinical effectiveness, provider adoption, and health system impact. Future research should focus on developing, implementing, and validating AI-assisted decision support tools and workflows for hepatic steatosis management. Randomized controlled trials comparing AI-assisted workflows to usual care could provide definitive evidence for improving outcomes and cost-effectiveness in MASLD management.

## Conclusion

Nearly 1 in 3 MASLD patients experienced identifiable management gaps, with high-risk patients exhibiting normal transaminases among the most concerning. These findings challenge current manual screening paradigms and highlight the need for systematic FIB-4-based risk assessment and streamlined referrals. AI-assisted clinical decision support and automated workflows offer a promising strategy to close care gaps and transition MASLD management to a proactive, risk-stratified model that consistently applies evidence-based guidelines to optimize patient outcomes.
